# Fatal fulminant community-acquired pneumonia caused by hypervirulent *Klebsiella pneumoniae* K2-ST86: Case report

**DOI:** 10.1097/MD.0000000000020360

**Published:** 2020-05-22

**Authors:** Hiroyuki Yamamoto, Anna Iijima, Kumiko Kawamura, Yasuo Matsuzawa, Masahiro Suzuki, Yoshichika Arakawa

**Affiliations:** a Department of Cardiovascular medicine, Narita-Tomisato Tokushukai Hospital, Chiba; b Pathophysiological Laboratory Sciences, Nagoya University Graduate School of Medicine, Aichi; c Department of Internal Medicine, Toho University Medical Center, Sakura Hospital, Chiba; d Department of Microbiology, Fujita Health University, Aichi; e Department of Bacteriology, Nagoya University Graduate School of Medicine, Aichi, Japan.

**Keywords:** community-acquired pneumonia, fatal fulminant pneumonia, hypervirulent *Klebsiella pneumoniae*, K2-ST86, multilocus sequencing typing

## Abstract

**Rationale:**

Invasive community-acquired infections, including pyogenic liver abscesses, caused by hypervirulent *Klebsiella pneumoniae* (hvKp) strains have been well recognized worldwide. Among these, sporadic hvKp-related community-acquired pneumonia (CAP) is an acute-onset, rapidly progressing disease that can likely turn fatal, if left untreated. However, the clinical diagnosis of hvKp infection remains challenging due to its non-specific symptoms, lack of awareness regarding this disease, and no consensus definition of hvKp.

**Patient concerns:**

A 39-year-old man presented with high-grade fever and sudden-onset chest pain. Laboratory testing revealed an elevated white blood cell count of 11,600 cells/μl and C-reactive protein level (>32 mg/dl). A chest X-ray and computed tomography revealed a focal consolidation in the left lower lung field.

**Diagnosis:**

Diagnosis of fulminant CAP caused by a hvKp K2-ST86 strain was made based upon multilocus sequencing typing (MLST).

**Interventions:**

The patient was treated with ampicillin/sulbactam.

**Outcomes:**

The pneumonia became fulminant. Despite intensive care and treatment, he eventually died 15.5 hours after admission.

**Lessons:**

This is the first case of fatal fulminant CAP caused by a hvKp K2-ST86 strain reported in Japan. MLST was extremely useful for providing a definitive diagnosis for this infection. Thus, we propose that a biomarker-based approach should be considered even for an exploratory diagnosis of CAP related to hvKp infection.

## 1 Introduction

*Klebsiella pneumoniae* is the main causative agent of respiratory and urinary tract infections that commonly occur in patients with impaired host defenses in healthcare settings. ^[1]^ However, pyogenic liver abscesses (PLA) caused by hypervirulent *K. pneumoniae* (hvKp) strains have emerged and have been reported mainly in East Asia since the 1980s.^[2]^ This strain can cause other community-acquired invasive infections, such as endophthalmitis, meningitis, and pneumonia, with high morbidity and mortality.^[3]^ Nowadays, hvKp infections are being increasingly recognized worldwide.

The hvKp strains are considerably more virulent than classical *K. pneumoniae* (cKp) strains and are characterized by a hypermucoviscous colony phenotype, capsular serotypes (K1/K2), and the production of virulence factors, including proteins encoded by K1-specific mucoviscosity-associated gene A (*magA*), K2-specific regulator of mucoid phenotype A gene (*rmpA/A2*), and siderophore-related genes, including ferric aerobactin receptor gene (*iutA*), yersiniabactin gene (*ybtS*), and gene for the outer membrane receptor, FepA, of Fe^3+^-bound salmochelin (*iroN*). ^[3,4]^ Moreover, multilocus sequencing typing (MLST) has allowed the molecular characterization of hvKp strains. While a majority of hvKp isolates are identified as K1-sequence type (ST) 23 to cause mainly PLA, genetically diverse K2 sequence types including ST65, ST86, ST375, and ST380, have been reported to cause various community-acquired invasive infections.^[5]^

Sporadic hvKp-associated community-acquired pneumonia (CAP) can be life-threatening if left undiagnosed. However, clinical diagnosis of such infections remains challenging because the PLA phenotype is less frequently detected and a consensus regarding the definition of hvKp is lacking. Therefore, a proper strategy to differentiate hvKp strains from cKp strains is needed.

Herein, we describe the first case of fatal CAP caused by a hvKp ST86 strain of serotype K2 in Japan. We also propose a new diagnostic strategy for identifying hvKp strains.

## 2 Case report

**Figure 1:**
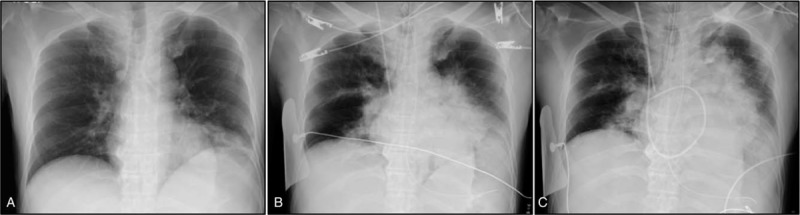
An initial chest X-ray revealed infiltration in the left lower lobe of the lung (A). Follow-up chest X-rays revealed a rapid progression to voluminous hyperdense lung infiltration with bulging interlobar fissure in the left lung and developing multiple patchy infiltrations in the right lung (B, 9 hours after admission and C, 11 hours after admission).

**Figure 2:**
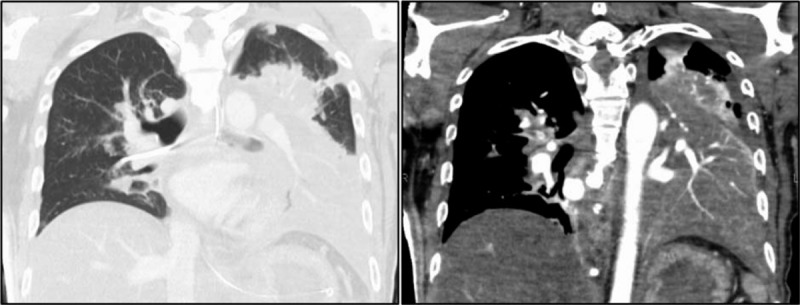
Chest computed tomography images without (left panel) and with (right panel) contrast enhancement. Note the enhanced pulmonary vasculatures within an airless low attenuating intense consolidation in the lower left lobe, known as a computed tomography angiogram sign, and multiple patchy infiltrates in both lobes.

A 39-year-old previously healthy Sri Lankan man living in Japan was admitted to our emergency department with fever and sudden-onset chest pain on the left side. He had no medical or travel history within the previous 6 months. Upon initial examination, the patient had a blood pressure of 153/111 mm Hg, high-grade fever of 38.0°C, tachycardia of 143 beats/minute, tachypnea of over 30 breaths/minute, and oxygen saturation of 96% on ambient air. However, no remarkable crackles were audible. Laboratory testing revealed a white blood cell count of 11,600 cells/μl with 84% neutrophil granulocytes, elevated levels of C-reactive protein (>32 mg/dl, reference: <0.3 mg/dl), 54 U/L aspartate aminotransferase (reference: 13–30 U/L), 66 U/L alanine aminotransferase (reference: 10–42 U/L), 438 U/L lactate dehydrogenase (reference: 124–222 U/L), 1277 U/L gamma-glutamyl transferase (reference: 13–64 U/L), and 291 mg/dl blood glucose (reference: <110 mg/dl). Urine antigen testing for *Streptococcus pneumoniae* and *Legionella pneumophila* gave negative results. Electrocardiogram revealed sinus tachycardia. A chest X-ray revealed a focal consolidation in the left lower lung field (Fig. 1A). The patient was hospitalized with CAP at 12:00 am on October 26th and promptly treated with ampicillin/sulbactam 1.5 g, every 6 hours. However, his condition rapidly deteriorated and he lost circulation at 8:43 am. Cardiopulmonary resuscitation was promptly initiated. The patient was intubated for mechanical ventilation. He achieved a return of spontaneous circulation at 8:56 am and was immediately transferred to the intensive care unit. He had a high Acute Physiology and Chronic Health Evaluation (APACHE) II score of 20 and a high lactic acidosis level (10.3 mEq/L, reference: 0.7–2.3 mEq/L). Follow-up chest X-rays revealed rapidly progressive infiltration following admission (Fig. 1B and 1C). Chest computed tomography demonstrated voluminous hyperdense infiltration with a bulging interlobar fissure in the left lower lobe, and multiple patchy infiltrates in both lung fields (Fig. 2), suggesting invasive pulmonary infection with septic emboli. Abdominal computed tomography showed severe fatty infiltration of the liver without abscess formation and pancreatic atrophy with multiple punctate calcifications and diffuse fatty replacement, suggesting chronic alcoholic change. Despite intensive treatment, the patient eventually died of multi-organ failure and disseminated intravascular coagulopathy caused by severe septic shock at 3:30 pm.

**Figure 3:**
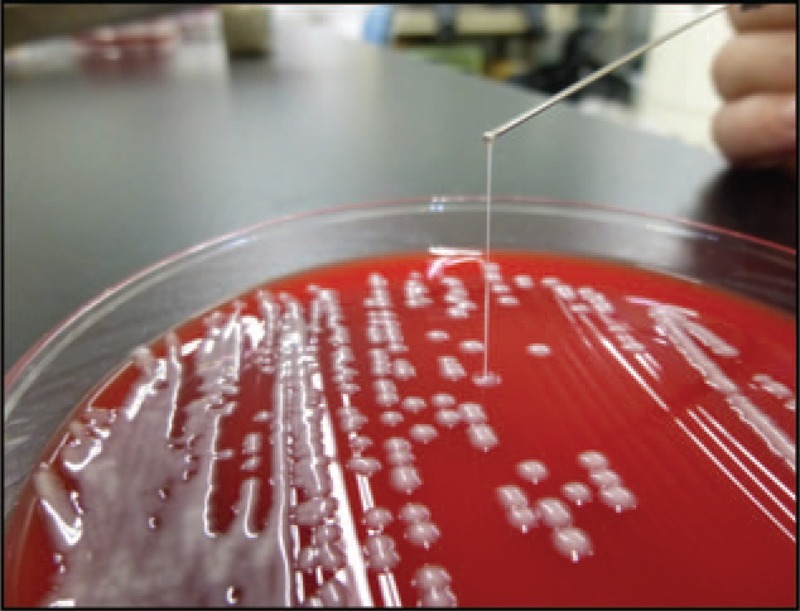
String test. The *K. pneumoniae* isolate recovered from our patient displayed a hypermucoviscous colony phenotype with a positive ultra-long viscous string >20 mm in length from a colony formed on a blood agar plate.

Later, sputum and blood cultures yielded *K. pneumoniae* that was susceptible to conventional antimicrobials, yet resistant to ampicillin. Moreover, the *K. pneumoniae* isolated was positive for the string test (Fig. 3). We performed capsular genotyping, PCR screening for virulence factors, and next-generation sequencing (NGS)-aided MLST of the isolate. The isolate was found to belong to an ST86 serotype K2 strain harboring virulence-associated genes including *rmpA*, *iutA*, *ybtS*, *iroN*, and type 3 fimbrial adhesin gene (*mrkD)*.

## 3 Discussion

To our knowledge, we describe here, the first case of fatal fulminant CAP caused by a hvKp K2-ST86 strain in Japan. Moreover, we demonstrated that MLST is effective for definitively detecting hvKp strains.

Several cases of severe CAP caused by hvKp K2-ST86 strains have been reported previously.^[6,7]^ A single-center study conducted in France demonstrated that hvKp, predominantly including K2-ST86 strains, account for 46% of severe CAP cases requiring intensive management, with a high mortality rate of 50%.^[8]^ The K2-ST86 *K. pneumoniae* isolate obtained from the patient whose case has been presented here caused CAP with a hypermucoviscous colony phenotype and carried *rmpA, iutA*, *ybtS*, and *iroN*, similarly to all hvKp strains known to cause severe CAP in France.^[8]^ Notably, *rmpA* has been significantly associated with the hvKp strain. ^[9]^ Moreover, aerobactin, a dominant siderophore produced by hvKp, is required for the in vivo growth of the bacterial pathogens and is considered a crucial factor for their hypervirulence.^[10]^ In addition, glucose intolerance, presumably due to chronic alcohol changes, might have affected the catastrophic phenotype in this case because hyperglycemia impairs the neutrophil phagocytosis of K1/K2 capsular serotypes.^[11]^

While the prevalence of K2-ST86 hvKp strains reported worldwide considerably varies (14%−46%), ^[12,13,14]^ these strains accounted for 15% to 25% of clinical isolates of *K. pneumoniae* of serotype K2 in Japan; yet, detailed clinical information regarding them is lacking.^[15,16]^ Given the large number of underdiagnosed fatal cases of hvKp infection that have a rapid progressive course and escape clinical recognition, severe CAP cases caused by K2-ST86 hvKp strains might be extremely prevalent. Moreover, the emergence of multidrug-resistant or carbapenem-resistant hvKp strains will pose serious threats to public health.^[17,18]^

Management of hvKp CAP includes parenteral antimicrobial regimens combined with sufficient source control including lung abscess and pleural empyema. Although these strains are usually highly susceptible, there are no randomized controlled trials evaluating the efficacy, dosage, and duration of various antimicrobials against hvKp infections. Given its high toxicity and its tendency to cause metastatic spread, an initial empirical antimicrobial regimen should be considered while waiting for the results of culture and susceptibility testing. Third-generation cephalosporins, fluoroquinolones or carbapenems with or without an aminoglycoside may be the favored antimicrobial regimen.^[19]^ In general, duration of antimicrobial therapy ranging from 4 to 6 weeks has been recommended. Periodic clinical and laboratory monitoring and follow-up imaging is also recommended to determine the response to therapy and the duration of antimicrobials. Otherwise, improper treatment may increase the recurrence rate.

Notably, the prognosis of hvKp CAP is considerably poor. A recent study conducted in Taiwan analyzed 93 patients with bacteremic CAP (49 due to hvKp and 44 to *Streptococcus pneumoniae*).^[20]^ The mortality rate was considerably higher in patients with bacteremic hvKp CAP (55.1 vs 27.3%). Also, an early mortality rate (death less than 48 hours after admission) was high (37.6 vs 13.6%). Septic shock and respiratory failure on initial examination are independent risk factors for fatal outcome in patients with bacteremic hvKp CAP. Importantly, clinicians should recognize the clinical importance of early diagnosis and prompt treatment because of the extremely high mortality rate in patients with hvKp CAP. However, the definition of hvKp remains uncertain. A hypermucoviscous colony phenotype, widely believed to be characteristic of hvKp strains, is not identical to hypervirulence.^[5]^ Capsular serotyping and string test are not enough to recognize hvKp strains due to a lack of accuracy. All the *rmpA*, *iutA*, and *iroN* genes identified in this case have recently been confirmed as critical biomarkers for accurately differentiating hvKp strains from cKp strains.^[21]^ This notion is supported by the fact that hvKp lineages, such as clonal groups 23, 86, and 380, carry virulence plasmids encoding all these genes.^[22,23]^ Thus, we propose that a biomarker-based approach utilizing MLST is highly reliable for identifying hvKp infections.

We described herein a fatal case of fulminant CAP caused by a hvKp K2-ST86 strain, which is the first case of its kind reported in Japan, to the best of our knowledge. We also demonstrated that MLST is remarkably useful for definitively diagnosing hvKp infections.

## 4 Ethics approval and consent to participate

Written consent was not obtained from the patient. Because no family members could not be contacted, the authorization for waiver of consent was approved by the Institutional Review Board (IRB) of Narita-Tomisato Tokushukai Hospital with the permission of the Director, Dr. Hidemitsu Ogino. All images in the current case are entirely unidentifiable and patient anonymity was completely preserved. The head of the IRB took responsibility for the anonymization of the patient.

## Acknowledgments

We express our sincere thanks to Prof. Kyoji Moriya and the technicians of the microbiology laboratory in the University of Tokyo Hospital for performing the initial PCR experiments for the *Klebsiella pneumoniae* isolate.

## Author contributions

**Conceptualization:** Hiroyuki Yamamoto.

**Data curation:** Hiroyuki Yamamoto, Yasuo Matsuzawa.

**Funding acquisition:** Yoshichika Arakawa.

**Investigation:** Anna Iijima, Kumiko Kawamura, Masahiro Suzuki.

**Supervision:** Masahiro Suzuki, Yoshichika Arakawa.

**Validation:** Anna Iijima, Kumiko Kawamura, Masahiro Suzuki.

**Writing – original draft:** Hiroyuki Yamamoto, Yoshichika Arakawa.
